# Supplementary material for: What does it take to learn the rules of RNA base pairing? A lot less than you may think
Source: bioRxiv. 2025 Aug 2:2025.07.31.668042. Preprint. [Version 1] doi: 10.1101/2025.07.31.668042 (PMC12324431; doi:10.1101/2025.07.31.668042)
Supplement: Supplement 1 [file media-1.gz › supplemental_material/experiments/g6_optimize_param_conus_rnabench_RNaseP_uniform_shuffle.B_shuffle/losses_i59.pdf]

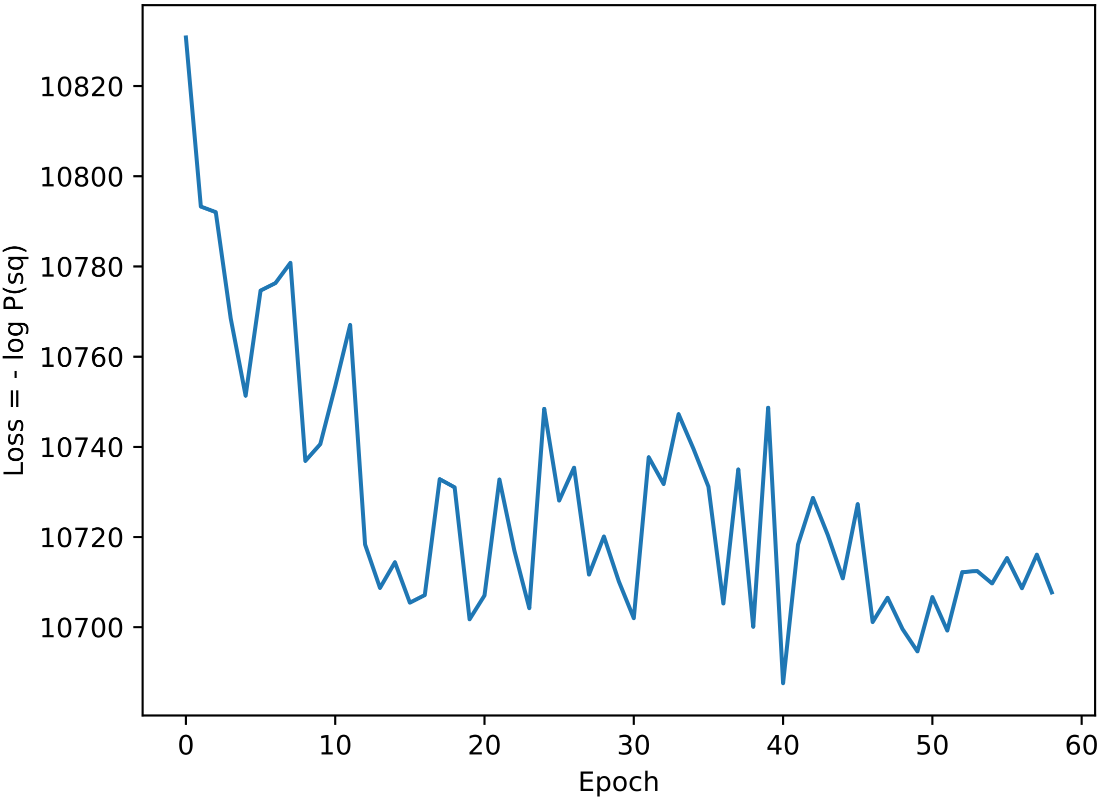

10820
10800
10780
10760
10740
10720
10700
Loss = - log P(sq)
0
10
20
30
40
50
60
Epoch
